# EZH2 in normal hematopoiesis and hematological malignancies

**DOI:** 10.18632/oncotarget.6198

**Published:** 2015-10-20

**Authors:** Laurie Herviou, Giacomo Cavalli, Guillaume Cartron, Bernard Klein, Jérôme Moreaux

**Affiliations:** ^1^ Department of Biological Hematology, CHU Montpellier, Montpellier, France; ^2^ Institute of Human Genetics, CNRS UPR1142, Montpellier, France; ^3^ University of Montpellier 1, UFR de Médecine, Montpellier, France; ^4^ Department of Clinical Hematology, CHU Montpellier, Montpellier, France

**Keywords:** hematological malignancies, EZH2, Polycomb complex, therapeutic target

## Abstract

Enhancer of zeste homolog 2 (EZH2), the catalytic subunit of the Polycomb repressive complex 2, inhibits gene expression through methylation on lysine 27 of histone H3. EZH2 regulates normal hematopoietic stem cell self-renewal and differentiation. EZH2 also controls normal B cell differentiation. EZH2 deregulation has been described in many cancer types including hematological malignancies. Specific small molecules have been recently developed to exploit the oncogenic addiction of tumor cells to EZH2. Their therapeutic potential is currently under evaluation. This review summarizes the roles of EZH2 in normal and pathologic hematological processes and recent advances in the development of EZH2 inhibitors for the personalized treatment of patients with hematological malignancies.

## PHYSIOLOGICAL FUNCTIONS OF EZH2

### EZH2 and Polycomb complex-mediated transcription repression

Epigenetic modifications play important biological roles because they regulate gene expression by modifying chromatin organization and by modulating transcription initiation, elongation, splicing and termination. Epigenetic modifications are dynamically established by DNA methyltransferases (DNMTs) and by many categories of histone-modifying enzymes that write, read and erase histone modifications [1] in a highly regulated manner. Given the importance of epigenetic information in the control of DNA functions, epigenetic deregulations play a key role in the development of many diseases, including cancer where they have been extensively studied [2-5].

EZH2, one of the most studied histone-modifying enzymes, is the catalytic subunit of Polycomb Repressive Complex 2 (PRC2). PRC2 is one of the two major classes of Polycomb complexes and is responsible for maintaining its target genes in a transcriptionally repressed state through tri-methylation of lysine 27 on histone H3 (H3K27me3) [6]. The other PRC2 members are EED, SUZ12, RBAP46/48 and AEBP2. Although their function has not been fully characterized, it is clear that EZH2 requires at least EED and SUZ12 to be catalytically active in vitro, while RBAP46/48 and AEBP2 stimulate EZH2 activity [6].

**Figure 1:**
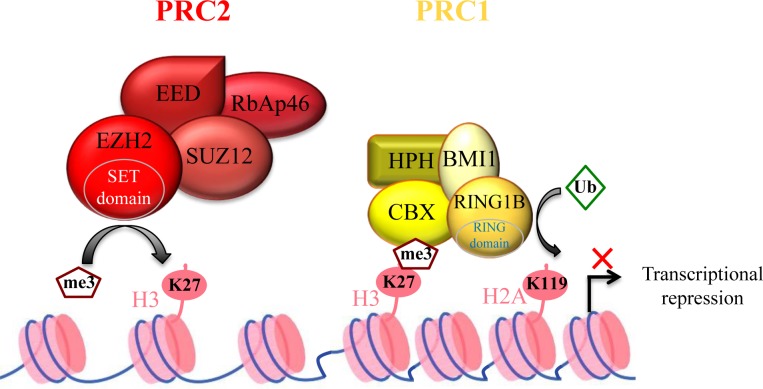
Polycomb complex-mediated transcription repression Polycomb Repressive Complex 2 (PRC2) contains four core components. EZH2 (Enhancer-of-zeste homolog 2) is the catalytic sub-unit, whereas EED (Embryonic Ectoderm Development), SUZ12 (zinc finger) and RBAP46/48 (Retinoblastoma Binding Protein) contribute to improving EZH2 activity. PRC2 induces EZH2-mediated H3K27 tri-methylation (H3K27me3) to repress its target genes. The H3K27me3 mark is recognized by the CBX subunit of Polycomb Repressive Complex 1 (PRC1). Then, the E3 ubiquitin-protein ligase RING1A ubiquitinylates histone H2A at lysine 119, strengthening gene transcription repression.

It has been suggested that EZH2-mediated H3K27me3 allows Polycomb Repressive Complex 1 (PRC1) recruitment to chromatin that will then mono-ubiquitinylate lysine 119 of histone H2A (H2AK119ub1), thus establishing a higher repressive state of chromatin (Figure 1) [7, 8]. Alternatively, following H2AK119ub1 by alternative PRC1-like complexes, PRC2 components could be recruited. They, in turn, will drive the formation of canonical PRC1, suggesting an intimate interplay between PRC1 and PRC2 for target gene silencing [9, 10].

### Roles of EZH2 in normal hematopoiesis

PRC1 and PRC2 play a major role in normal hematopoiesis by promoting pluripotency maintenance and self-renewal of adult stem cells [8, 11]. EZH2 involvement in hematopoiesis was first revealed by its interaction with VAV [18], an important effector in hematopoietic signal transduction [19].

As EZH2, EED or SUZ12 loss-of-function mutations increase the activity of hematopoietic stem cells (HSCs) and progenitor cells, PRC2 could contribute to their negative control [20]. EZH2 overexpression in HSCs prevents exhaustion of their long-term repopulating potential during serial transplantation [18, 21, 22]. Therefore, EZH2 could prevent stem cell senescence [18] by regulating adult HSC differentiation, but not their self-renewal capacity [22-24]. EZH2 also increases the pool of quiescent HSCs by supporting their proliferation and inhibiting apoptosis. However, EZH2 inactivation has opposite effects on apoptosis and proliferation in multi-potent progenitors compared with long-term HSCs (LT-HSCs) [23]. In LT-HSCs, EZH2 is associated with hematopoietic differentiation and function, cellular growth and proliferation and survival [24].

One of the critical issues for understanding the role of EZH2 in hematopoiesis is to find, among the thousands of genes bound and modified by PRC2, the key targets of its transcriptional regulation. CDKN2A is one of the most important Polycomb target loci in mammals. CDKN2A negatively regulates cell proliferation through the RB/p53 pathway and is repressed by EZH2. However, EZH2 requirement for HSC proliferation and differentiation is not only dependent on *CDKN2A* repression, suggesting that other PRC2 target genes must be involved in this function [24]. Indeed, EZH2 also regulates cell cycle genes to promote cell cycling and controls the expression of genes that inhibit HSC differentiation (*ID2* and *SOX7*) [24, 25]. Moreover, EZH2, by repressing pro-apoptotic genes, such as *NOXA*, *p21* and *WIG1*, protects HSCs from cell death [24].

During lymphopoiesis, EZH2 is strongly expressed in proliferating cells, such as human germinal center B cells, cycling T and B lymphocytes and plasmablasts, suggesting an important role in cell cycle regulation and in lymphocyte division [25-27]. In agreement with this hypothesis, lower levels of H3K27me3 and other histone methylation marks are observed in resting B-cells compared with activated and cycling cells. The histone H3.3 variant accumulates in non-cycling cells and directly reflects the length of time lymphocytes remain quiescent [28]. This suggests that canonical H3K27me3 could be replaced by the H3.3 variant that is not methylated at K27 [29, 30].

On the other hand, EZH2 is down-regulated during B-cell differentiation and maturation [27]. The authors of this study suggested that EZH2 is a component of the checkpoint mechanism that controls pro-B to pre-B cell transition [27]. EZH2 regulates pre-B cell receptor (pre-BCR) expression in pro-B cells. This receptor is essential for B-cell maturation, by directing both cell cycle exit and Igκ recombination at the pre-B cell stage. It has been proposed that H3K27me3 mark on the VhJ558 gene allows the specific targeting of the recombination machinery, DNA cleavage and gene rearrangement in pro-B cells [27].

Another study suggested that EZH2 could inhibit pro- to pre-B cell differentiation by interacting with STAT5. Activation of the IL7 receptor and STAT5 would lead to EZH2 recruitment, repression of Igκ transcription and inhibition of its recombination [31]. STAT5 and EZH2 also act together to repress a significant subset of genes during B-cell lymphopoiesis [31]. Moreover, EZH2 controls B-cell differentiation and immunoglobulin heavy chain (IgH) gene rearrangement during mouse B-cell development [21, 22].

During B-cell differentiation, EZH2 also blocks DNA repair response pathways in activated B-cells, thus allowing their survival following Activation-Induced cytidine Deaminase (AID)-mediated somatic hypermutation of Ig genes [32] (Figure 3).

## EZH2 AND HEMATOLOGICAL MALIGNANCIES

During the last ten years, EZH2 has generated much interest as a potential therapeutic target in cancer. First, EZH2 overexpression was correlated with tumor cell aggressiveness, metastasis development and poor prognosis in different cancer types [12-14]. More recently, EZH2 gain-of-function somatic mutations have been discovered [15-17]. These results led academic laboratories and pharmaceutical companies to develop molecules to target EZH2.

### Increased EZH2 expression

A number of studies have shown that *EZH2* overexpression plays a major role in the physiopathology of several hematological malignancies by promoting cell proliferation and inducing tumor cell phenotypes. High *EZH2* expression in lymphomas is correlated with increased proliferation, tumor cell aggressiveness and poor prognosis [33, 34]. *EZH2* is also overexpressed in 100% of Burkitt lymphomas (BL), 87.5% of grade 3 follicular lymphomas (FL) and 85.7% of diffuse large B-cell lymphomas (DLBCL) [33].

Similarly, *EZH2* overexpression in natural killer (NK)/T-cell lymphomas is associated with a growth advantage and poor prognosis [35]. *EZH2* is also strongly expressed in mantle cell lymphomas where high *EZH2* expression is correlated with aggressiveness and poor prognosis [36, 37]. Increased *EZH2* expression in these malignancies is partly due to MYC-mediated inhibition of miR-26 and miR-101, two microRNAs that target EZH2 [35, 38].

In the B-cell acute lymphoblastic leukemia (ALL) Nalm-6 cell line, *EZH2* overexpression is correlated with silencing of tumor suppressor genes (p21, p53 and PTEN) [39]. More recently, it has been reported that *EZH2* is significantly overexpressed in tumor cells from patients with chronic lymphoid leukemia compared with paired healthy B-cells [40]. This increased expression is correlated with hyper-lymphocytosis, chromosomal abnormalities, high ZAP-70 expression and poor prognosis [40].

In several cancers, *EZH2* expression is significantly correlated with expression of *MMSET,* a histone methyltransferase that regulates transcription and oncogenesis in multiple myeloma (MM) harboring t(4;14) translocations [41]. EZH2 positively regulates *MMSET* expression at the post-transcriptional level by repressing the expression of miR-26a, miR-31 and miR-203, thus promoting tumor development [41]. Moreover, *MMSET-*overexpressing cells are more sensitive to EZH2 inhibitors [42].

Other studies support a potential role for EZH2 in MM pathogenesis, but the underlying mechanisms are still poorly understood. Global gene expression profiling indicated that *EZH2* is up-regulated in monoclonal gammopathy of undetermined significance (MGUS) and aggressive myeloma cells compared with normal plasma cells [43]. Moreover, H3K27me3 target genes are down-regulated in MGUS and MM compared with normal bone marrow plasma cells [44]. In human MM cell lines (HMCL), *EZH2* expression has been correlated with proliferation and growth factor independence [45]. Moreover, *EZH2* expression is required in HMCL with activated N-RAS and K-RAS proliferative phenotypes [45]. Inhibition of EZH2 expression and activity, and thus loss of Polycomb target gene repression, is associated with HMCL growth inhibition [46, 47] and decreased tumor load and survival in a mouse model of MM [44]. Although these effects were correlated with decreased EZH2 expression, the inhibitors used in these studies were not specific [48]. Therefore, specific inhibitors of EZH2 enzymatic activity are required to precisely define its roles in MM. Finally, one study reported that *EZH2* is overexpressed in the side population cells of MM cell lines. These side population cells, which are characterized by their ability to export Hoechst dye and by CD138 expression, actively proliferate and show tumor-initiating potential [49].

Altogether, these studies suggest an involvement of EZH2 in MM initiation/development and aggressiveness, but its role in this malignancy still need to be fully characterized.

A correlation between *EZH2* overexpression and myeloid leukemia development has also been described [50]. *EZH2* is highly expressed in high-risk myelodysplastic syndrome (MDS) and in acute myeloid leukemia (AML) arising from a pre-existing MDS. Specifically, *EZH2* is significantly overexpressed in MDS and AML primary tumor cells that display aberrant DNA methylation of the gene encoding the tumor suppressor p15INK4B compared with tumors where p15INK4B is not methylated [51].

In mice, *Ezh2* overexpression in HSCs leads to myeloproliferative neoplasms (MPNs) [23]. In this model, several genes associated with HSC maintenance are regulated by EZH2, including the transcriptional regulators *Evi-1* and *Ntrk3* that are aberrantly expressed in hematological malignancies [23]. In another AML murine model, *Ezh2* loss inhibits cancer cell proliferative capacity and disrupts tumor progression by re-activating EZH2 target genes that are implicated in myeloid cell differentiation [52]. These results obtained in several mouse models highlight EZH2 oncogenic function in myeloid cells.

*EZH2* overexpression in cancer cells may result from different mechanisms. *EZH2* up-regulation has been linked to gene amplification in several solid cancers [53], but not in hematological malignancies. Alternatively, increased expression and/or activity of *EZH2* positive regulators could lead to *EZH2* overexpression. For instance, in hematological malignancies, c-MYC is deregulated by different mechanisms, including chromosome rearrangements, amplification, mutations and also epigenetic mechanisms, such as aberrant microRNA regulation [54-59]. Enhancement of *EZH2* expression by c-MYC has been described in prostate cancer and AML [60, 61]. Therefore, a link between *c-MYC* deregulation and *EZH2* overexpression could be involved in the physiopathology of hematological malignancies. On the other hand, overexpression of the ETS DNA binding protein ERG in a CML cell line is associated with drug resistance and repression of DNA chromatin remodeling genes, including the PRC2 members *SUZ12* and *EZH2* [62]. Other transcription factors, such as HIF1α, ELK1, EWS-FLI1, E2F, ATAD2/ANCCA, NF-YA, STAT3 and BRAF, can modulate *EZH2* gene expression in solid cancers [63-73]. As their expression/activity is also deregulated in hematological malignancies, it could be important to analyze their role in normal hematopoiesis and during tumorigenesis.

### Somatic mutations leading to EZH2 gain of function

**Figure 2:**
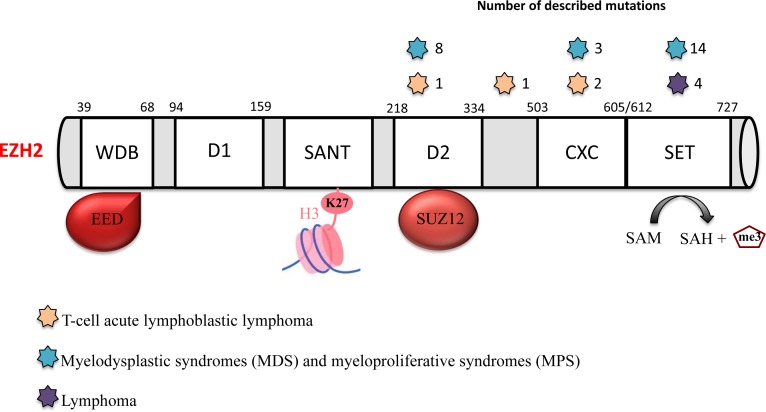
EZH2 mutations in hematological malignancies EZH2 has several functional domains: the WD-40 binding domain (WDB) that interacts with EED; domain 1 (D1); domain 2 (D2) that interacts with SUZ12; the domains SWI3, ADA2, N-CoR and TFIIIB (SANT) that interact with histones; a cysteine-rich domain (CXC); and the suppressor of variegation 3-9, enhancer of zeste and trithorax (SET) catalytic domain. Several EZH2 mutations have been identified in different hematological malignancies: lymphoma (purple star), myelodysplastic syndromes (MDS) and myeloproliferative syndromes (MPS) (blue stars) and T-cell acute lymphoblastic lymphoma (T-ALL) (orange stars).

**Figure 3:**
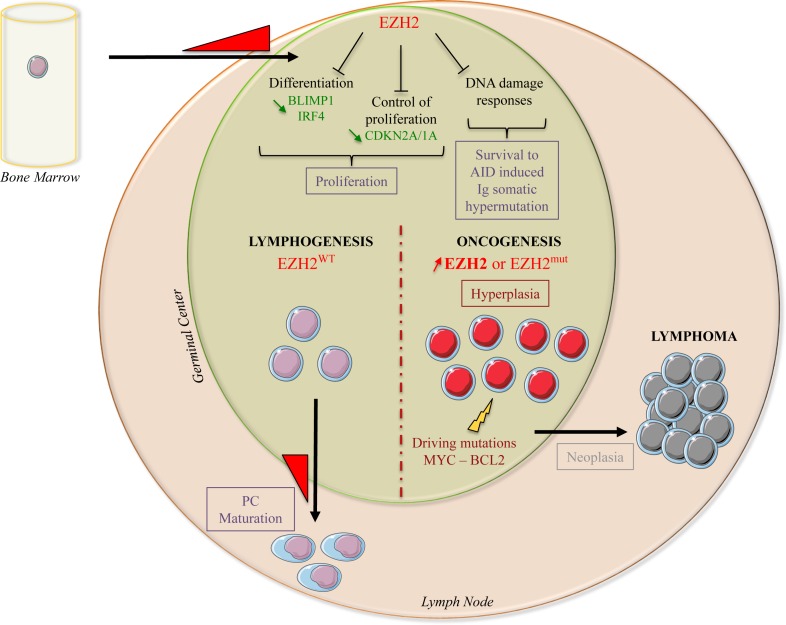
EZH2 biological functions during normal B-cell differentiation and lymphomagenesis During normal B-cell differentiation, *EZH2* expression is induced when naive B-cells enter the germinal center for affinity maturation and antibody class switching. Through H3K27 tri-methylation, EZH2 represses the expression of negative cell cycle regulators (*CDKN2A* and *CDKN1A*) and of B- cell differentiation transcription factors (*BLIMP1* and *IRF4*), leading to immature B-cell expansion. EZH2 also inhibits DNA damage response pathways, allowing survival of B-cells during AID-mediated somatic hypermutation of Ig genes. *EZH2* expression decreases when B-cells exit the germinal centers, leading to de-repression of EZH2 target genes to support plasma cell maturation. Conversely, activating mutations or EZH2 overexpression lead to an imbalance due to increased repression of EZH2 target genes, induction of proliferation and blockade of B-cell maturation. Additional oncogenic hits (c-MYC or BCL2 deregulation) will then further promote cell transformation.

Recurrent heterozygous mutations that target specifically Y641 in the SET catalytic domain within the S-adenosylmethionine (SAM) pocket of EZH2 were described in germinal center B-cell diffuse large B-cell lymphoma (GCB DLBCL) and in FL and are found in 22% of patients [17]. Other recurrent somatic mutations were identified at A677 and A687 of the EZH2 SET domain [15-17, 74] (Figure 2). These mutations are less frequent and are detected only in 1 to 2% of patients with DLBCL or FL. Unlike wild type EZH2 that preferentially targets non-methylated or mono-methylated lysine 27 of H3 (H3K27 or H3K27me1), EZH2^Y641^ mutants preferentially target di-methylated lysine 27 of H3 (H3K27m2), and have a weaker activity on H3K27 and H3K27me1 [16, 75]. The EZH2^A677G^ mutant methylates H3K27, H3K27me1 and H3K27me2, whereas EZH2^A677V^ targets preferentially H3K27me1 [16]. In agreement, lymphoma cell lines harboring EZH2^Y641^ or EZH2^A677^ display significantly higher H3K27me3 and lower H3K27me2 levels [16, 75]. Both histone modifications lead to inhibition of target gene expression, although it was suggested that H3K27me3 is associated with a more repressive chromatin state than H3K27me2 [76, 77]. How a decrease in H3K27me2 level contributes to the biology of EZH2-mutated lymphoma cells is not known. Although EZH2 inhibition induces the loss of di- and tri-methylation in all characterized lymphoma cell lines, independently of their *EZH2* mutational status, cell lines with EZH2 activating mutations that lead to higher H3K27me3 are more sensitive to EZH2 inhibitors than cells harboring wild type EZH2 [16, 78]. As H3K27me3 is a repressive mark, EZH2 inhibition in sensitive cell lines leads to expression of H3K27me3 target genes. Surprisingly, in cell lines that are not responsive to EZH2 inhibition, there are very few transcriptional changes, although H3K27me2/3 is completely lost [16]. These results suggest that inhibition of H3K27 methylation is not sufficient on its own to induce target gene transcription in unresponsive cell lines, and that other factors are implicated. Therefore, it would be interesting to target redundant epigenetic repressive marks (such as DNA methylation, H3K9 di- and tri-methylation), or the absence of activation marks, such as H3K4 tri-methylation or H3K27 acetylation (H3K27ac), to sensitize tumor cells to EZH2 inhibition. For instance, in DLBCL harboring EZH2 gain-of-function mutations, the cytotoxic and cytostatic effects induced by a histone deacetylase 1/2 (HDAC1/2) inhibitor were correlated with increased H3K27ac at genes involved in the DNA damage response or apoptosis. This treatment did not induce loss of H3K27me3 globally, but only at genes involved in the DNA damage response, thus leading to DSB repair impairment and apoptosis. This study suggests that EZH2 gain-of-function mutations represent a survival advantage for DLBCL cells by improving DNA damage repair processes [79]. In mice, the presence of EZH2^Y641^ mutations is correlated with higher number of germinal center B cells, germinal center hyperplasia and lymphoid transformation. This phenotype is associated with aberrant *EZH2* expression in tumor cells [80]. EZH2 deregulation interferes with the finely regulated balance between germinal center B cell proliferation and differentiation, blocking them in a proliferative and immature state, which constitutes a key determinant in lymphoma development [32, 80]. EZH2 gain-of-function mutations are not sufficient on their own for lymphoma development; however, they represent a driving hit in lymphoid transformation, in association with the deregulation of other genes, such as c-*MYC* or *BCL2* [80, 81] (Figure 3).

### Somatic mutations leading to EZH2 loss of function

On the other hand, several studies have shown that H3K27me3 inhibition also contributes to the development of hematological malignancies [83, 84]. This inhibition can be directly caused by EZH2 inactivating mutations, or indirectly by ASKL1 mutations that lead to loss of PRC2-mediated gene repression [82]. These data suggest that EZH2 can act not only as an oncogene, but also as a tumor suppressor, depending on the cell context. Two studies reported the presence of different EZH2 inactivating mutations in myeloid hemopathies, such as myelofibrosis, chronic myelomonocytic leukemia (CML), atypical chronic myelogenous leukemia and MDS [83, 84]. EZH2 inactivating mutations are associated with significantly shorter overall survival and event-free survival in these patients. EZH2 loss-of-function mutations were correlated with poor prognosis also in a cohort of patients with myelofibrosis [85]. A recent model suggests that EZH2 inactivating mutations contribute to cancer stem cell development through the induction of *HOXA9* expression, thus supporting myeloid progenitor self-renewal [86]. In MDS, EZH2-inactivating mutations are frequently associated with *RUNX1* mutations. In a MDS mouse model induced by a *Runx1* mutation in HSCs, *Ezh2* loss promotes MDS development, but reduces its propensity to progress to AML [87]. EZH2-inactivating mutations are not restricted to myeloid hemopathies. Indeed, *EZH2* or *SUZ12* mutations are also observed in 25% of patients with T-cell acute lymphoblastic leukemia (T-ALL) [88]. Several studies demonstrated that EZH2 loss-of-function mutations contribute to the development of T-ALL, mostly through activation of the NOTCH signaling pathway. Moreover, in mice injected with T-ALL cells, EZH2 inhibition is associated with significantly higher tumorigenic potential and mortality [88, 89].

Many studies on the involvement of Polycomb group proteins in cancer initiation and development have highlighted their dual role as oncogenes/tumor suppressors [15-17, 35, 83-88]. Accumulating evidence shows that PRC2 inhibits the proliferative and self-renewal potential of immature lymphoid progenitors [22-24] and that enhanced EZH2 activity specifically stimulates mature cell proliferation [25-27]. Therefore, it could be hypothesized that Polycomb group proteins act as oncogenes in differentiated cells, such as B, NK or NKT cells [32, 80, 81], and as tumor suppressors in undifferentiated cells, such as HSCs/progenitor cells [87]. Experimental modulation of EZH2 expression in a cell-stage specific manner during hematopoiesis might help validating this hypothesis and improving our understanding of EZH2 role in hematological malignancies.

### EZH2 functions not related to H3K27 methylation

The growth advantage associated with increased *EZH2* expression in NK/T-cell lymphoma cells is independent of its H3K27 methylation activity. Indeed, overexpressed EZH2 deregulates tumor cell proliferation by directly inducing *CCND1* transcription [35]. This H3K27me3-independent function in transcriptional activation has also been observed in breast and prostate cancer [90-92], suggesting a possible switch of EZH2 from silencer to activator during tumorigenesis in some cancers.

Concerning its silencing role, in addition to deposition of H3K27me3, the PRC2 complex can recruit HDACs and DNMTs that enhance its transcriptional repression activity. Treatment with HDAC inhibitors results in *EZH2* expression down-regulation in tumor cells, suggesting that PRC2 and HDACs collaborate in the epigenetic control of gene expression [93]. EZH2 can also directly interact with DNMTs [94]. Analysis of the DNA methylation profile of lymphoma cells revealed a significant enrichment in Polycomb complex target genes [95]. These data indicate that H3K27me3 and DNA methylation cooperate in repressing PRC2 target genes associated with lymphomagenesis.

## TARGETED EPIGENETIC THERAPY: EZH2 INHIBITORS

Considerable efforts have been focused on the development of EZH2 inhibitors. 3-Deazaneplanocin (DZNep) was the first molecule developed to inhibit EZH2 through the competitive inhibition of the enzyme S-adenosylhomocysteine hydrolase. This results in the accumulation of adenosylhomocysteine that inhibits methyltransferases and induces PRC2 degradation, thus leading to a decrease in EZH2 protein level [48]. As DZNep is a broad-spectrum inhibitor of methyltransferases, it is not considered a proper EZH2 targeted therapy [48]. Studies carried out in mice reported that DZNep could be of therapeutic interest as immunomodulator for the treatment of graft versus host disease (GvHD), underscoring the interest for allograft protocols in hematological malignancies [96]. Specifically, histone methylation can modulate the acquired immune response and DZNep activates the pro-apoptotic program in alloantigen-activated T-cells in a GvHD mouse model, without any effect on the antitumor activity of the donor T-cells.

The discovery of EZH2 mutations, particularly in DLBCL, promoted research to develop specific EZH2 inhibitors [16, 78, 97, 98], such as El1. E11 is recruited to wild type EZH2 or to Y641 mutants [98], resulting in H3K27me3 inhibition, arrest in the G1 phase of the cell cycle, apoptosis induction and differentiation of EZH2-mutated B-cells into memory B-cells. Two other EZH2 inhibitory molecules (EPZ005787 and GSK126) were identified independently by high-throughput screening [16, 78]. These two highly specific EZH2 inhibitors (500 to 1000 times more selective for EZH2 than for other methyltransferases) target EZH2 through the same mechanism as El1, thus inhibiting H3K27 tri-methylation and allowing the transcriptional activation of EZH2 target genes. The milder apoptosis induction in wild type lymphoma cell lines compared with EZH2^Y641^ cells (where EZH2 catalytic activity is stronger) provided the proof of concept of the efficiency of these inhibitors [16]. In a xenograft mouse model of DLBCL, the GSK126 inhibitor leads to tumor growth inhibition and significantly longer survival in treated mice compared with untreated controls [16].

A phase I/II multicenter clinical trial on the use of an EZH2 inhibitor as monotherapy (E7438 or Tazemetostat) in patients with B-cell lymphoma (GCB and non-GCB DLBCL and grade 3 FL) or with advanced solid cancers is currently ongoing (NCT01897571). Preliminary results showed an acceptable safety profile in all treated patients and objective responses in patients with relapsed DLBCL and FL harboring, or not, EZH2 mutations. Partial responses were observed as early as two months and up to ten months after the beginning of treatment, and time-dependent responses were reported (ClinicalTrial.gov reference NCT01897571).

The efficiency of targeted approaches in patients with tumors harboring EZH2-activating mutations can be improved by using companion diagnostic tests to identify patients with EZH2^Y641^ mutations [99]. Moreover, it could be interesting to associate EZH2 inhibitors with other inhibitors that target oncogenic pathways involved in hematological malignancies. For instance, recent *in vitro* studies have highlighted the synergy between EZH2 inhibitors and HDACi in aggressive B-cell lymphoma [38, 97]. These effects are mainly mediated by *EZH2* deregulation and MYC-induced microRNA inhibition. Moreover, as EZH2 and DNMTs cooperate in transcriptional regulation, it might be interesting to explore the effects of associating EZH2 inhibitors with DNA demethylating agents in B-cell lymphoma cells. Finally, it has been reported that the combination of the EZH2 inhibitor E7438 with a corticosteroid has an anti-proliferative effect in GCB-like lymphoma cell lines and sensitizes EZH2 inhibitor-resistant cell lines, independently of EZH2 mutational status [100].

## CONCLUSION AND PERSPECTIVES

**Figure 4:**
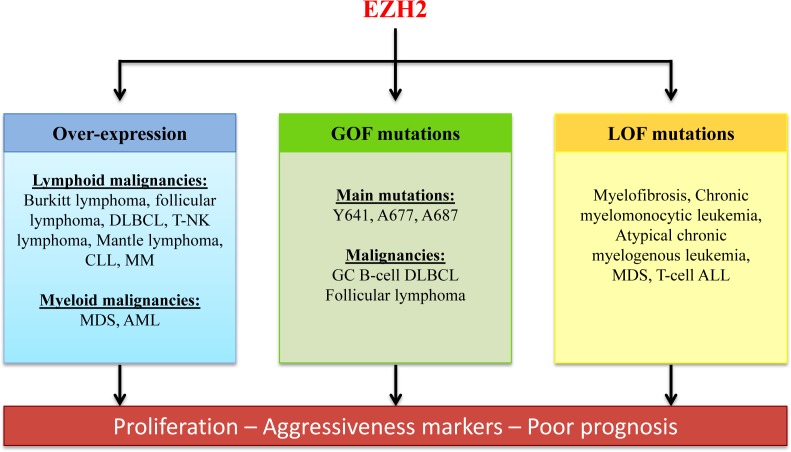
Role of EZH2 deregulation in hematological malignancies EZH2 is deregulated in several hematological malignancies. EZH2 overexpression and gain-of-function mutations are correlated with increased proliferation of tumor cells and poor prognosis in patients with lymphoma. However, EZH2 loss-of function mutations also can be associated with aggressiveness of some hematological malignancies. Therefore, therapeutic molecules targeting EZH2 should not hinder EZH2 tumor-suppressor function. GC B-cell DLBCL: Germinal center B-cell diffuse large B-cell lymphoma; CLL: chronic lymphocytic leukemia; MM: multiple myeloma, MDS: myelodysplastic syndrome; AML: acute myeloid leukemia; ALL: acute lymphocytic leukemia.

Significant advances have been made in understanding the epigenetic landscape in hematological malignancies, including the characterization of EZH2 biological functions. In some cancers, such as GCB lymphoma, EZH2 plays a key oncogenic role and represents a promising therapeutic target (Figures 3 & 4). In other tumor types, EZH2 can also have a tumor suppressor activity. Further studies are required to elucidate this duality between oncogenic and anti-tumor activities, depending on the cancer type and the cellular context (Figure 4).

The first clinical trials are currently ongoing to determine the safety and efficiency of EZH2 inhibitors. Preliminary clinical data in patients with DLBCL and FL indicate limited adverse events and a time-dependent objective response after monotherapy with Tazemetostat. Moreover, H3K27 methylation status and EZH2 expression could be used as prognostic markers to help developing personalized medicine in hematological malignancies. Specifically, a H3K27me3/H3K27me2 score combined with EZH2 mutational status has been proposed for selecting patients with DLBCL who could benefit from treatment with EZH2 inhibitors [101].

Moreover, the discovery of non-histone EZH2 substrates and of PRC2-independent EZH2 functions opens new research directions and will probably influence the development of a second generation of EZH2 inhibitors.
